# Use of Supraclavicular Flap by End to Side Technique in Pharyngeal SCC: A Case Report and Review of Literature

**DOI:** 10.1155/2021/6619916

**Published:** 2021-07-14

**Authors:** Aslan Ahmadi, Ayda Sanaei, Delaram Jan, Maryam Zolfaghary

**Affiliations:** ^1^ENT and Head & Neck Research Center, The Five Senses Institute, Iran University of Medical Science, Tehran, Iran; ^2^ENT and Head & Neck Research Center and Department, Five Senses Health Research Institute, Hazrat Rasoul Akram Hospital, Tehran, Iran

## Abstract

**Objectives:**

In recent years, conservation laryngeal surgeries, including partial pharyngectomy, have been introduced as an alternative procedure for selected cases of hypopharyngeal squamous cell carcinoma (HSCC). Reconstruction of these defects presents a considerable challenge for the surgeon after partial pharyngectomy due to its circumferential nature. In this case report, we represent the innovative “End to side” technique to reconstruct hypopharyngeal defect using the rolled supraclavicular flap after laryngeal-preserving partial pharyngectomy.

**Methods and Results:**

A 70-year-old female presented with a history of progressive dysphagia and odynophagia. The evaluations revealed a T3N0M0 SCC of pyriform sinus. The mass was successfully resected through partial pharyngectomy, and the hypopharyngeal defect reconstruction was achieved using the rolled supraclavicular flap via the “End to side” technique. The patient was discharged after decannulation on day 10. The 3-week barium swallow was performed with no evidence of anastomotic leakage, and the oral feeding was started after NG tube removal. At week 5, complete movement of the true vocal cord on the one side and good phonation and deglutition was observed. There was no evidence of recurrence after 1 year.

**Conclusions:**

Laryngeal-preserving partial pharyngectomy and hypopharyngeal reconstruction with the rolled supraclavicular flap via the “End to side” technique could lead to good oncological and functional outcomes in selected cases of pyriform sinus.

## 1. Introduction

Hypopharyngeal squamous cell carcinoma (HSCC) is an aggressive lesion, presenting the worst prognosis among head and neck cancers with a 5-year survival of approximately 30% [1–4]. Patients usually present with an advanced tumor due to submucosal spread, leading to minimal preliminary manifestation, but early lymph node involvement and distant metastasis [5, 6]. Thus, most patients require radical resection (i.e., total pharyngolaryngoesophagectomy (PLE)) resulting in high morbidity and mortality rate (e.g., permanent tracheostomy leading to phonation dysfunction and impaired swallowing) [7–9]. Recently, chemoradiotherapy treatment (CRT) has gained popularity as the primary treatment of HSCC, although it has several limitations mainly including a high recurrence rate, late toxicity, and short disease-free interval [10–12].

In recent years, conservative surgeries with laryngeal preservation protocols have been introduced as an alternative procedure for selected cases of invasive HSCC [13, 14]. Partial pharyngectomy, indicated in lesions of the pyriform sinus, is one of these procedures, leading to desirable postoperative oncologic and functional outcomes [15, 16] and a 5-year overall survival rate, disease-specific survival rate, and successful laryngeal function preservation of 50, 65%, and 80%, respectively [17].

Reconstruction of the hypopharyngeal defect represents a considerable challenge for the surgeon after partial pharyngectomy due to its circumferential nature [18]. The ideal reconstruction would lead to normal deglutition, phonation, and breathing [19, 20]. Several techniques have been described in the literature regarding the reconstruction of the hypopharynx including primary closure, regional flaps, free graft, and gastric pull-up [21]. The gastric pull-up is the traditional reconstructive method which has several vital disadvantages, such as high morbidity rate, the need for a second surgeon, and prolonged hospital stay [22]. Recently, the supraclavicular flap has emerged as an efficient and reliable flap choice for various head and neck defects [23].

Unfortunately, few articles focus on the larynx-preserving pharyngectomy approach as a primary treatment of HPSCC, and most of them performed total pharyngectomy or included cases with early tumors [24–27]. In our previous study, we described a new technique called “End to side” in patients with the SCC of the pyriform sinus apex undergoing partial pharyngectomy with good short-term and long-term results, in which we advanced and sutured the whole circumference of the mucosal part of esophageal inlet to cover pharyngeal wall defect to avoid postoperative stricture or dysphagia [28]. In this case report, we present the result of using this innovative technique to reconstruct hypopharyngeal defect with the rolled supraclavicular flap after laryngeal-preserving partial pharyngectomy.

## 2. Clinical Presentation

**Figure 1:**
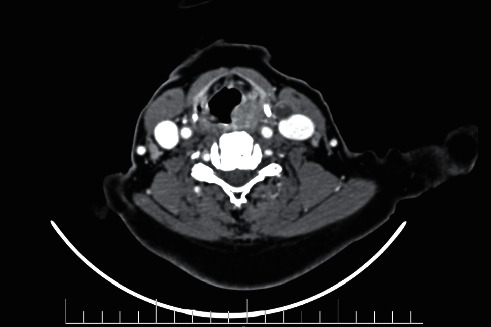
CT scan, axial view. There is an abnormal enhancing mass in the left pyriform sinus.

A 70-year-old female was admitted to our otorhinolaryngology clinic with a history of progressive dysphagia and odynophagia with neither hoarseness nor respiratory symptoms. The patient underwent direct laryngoscopy as a retropharyngeal mass was revealed involving the apex and lateral wall of the left pyriform sinus accompanied by circumferential involvement of the cervical esophagus, sparing the postcricoid area. A biopsy was taken from the lesion, and the histopathologic result was suggestive of SCC lesion. The preoperative staging was T3N1M0 based on the American Joint Committee on Cancer (AJCC) TNM system (Figure 1).

We obtained informed consent from the patient for all diagnostic and therapeutic procedures. The patient underwent partial pharyngectomy, cervical esophagectomy, total thyroidectomy, and bilateral neck dissection of levels 2–4.

**Step 1 .**

Using the apron incision, we approached the hypopharynx laterally; preserving the larynx, we resected the retropharyngeal wall to the level of the hyoid bone, left lateral pharyngeal wall, and pyriform sinus in a circumferential manner to the inlet of the esophagus; the left recurrent laryngeal nerve was sacrificed due to tumoral invasion. The mass was successfully excised with a lateral margin of 2 cm, an inferior margin of 1.5 cm, and a superior margin up to the epiglottis tip that left us with right piriform sinus, right lateral pharyngeal wall, a strip of retropharyngeal wall in the posterior, and esophageal lumen in the distal part.

**Step 2 .**

**Figure 2:**
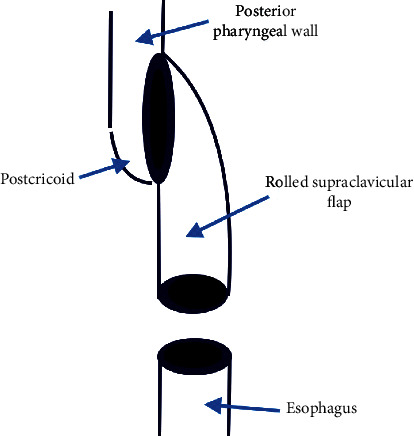
The rolled flap was advanced and connected (using modified Gambee suture) to pharyngeal wall to provide additional mucosa to cover the defect.

**Figure 3:**
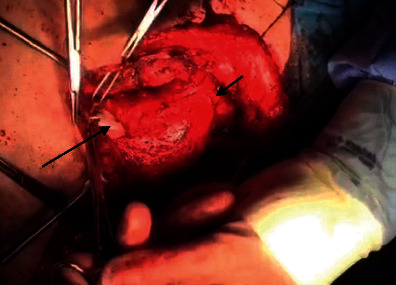
Short arrow indicates anastomosis of flap to oropharynx in proximal. Long arrow indicates anastomosis of rolled flap to cervical esophagus in distal part of the defect.

After harvesting the supraclavicular fasciocutaneous flap, we rolled it into an 8 cm tube to reconstruct the circumferential surgical defect (Figures 2 and 3).

**Step 3 .**

We linked the distal part of the rolled flap to the proximal part of the cervical esophagus using modified Gambee suture and then attached the other end of the tube to the lateral side of oropharyngeal and postcricoid mucosa from the left, applying the “End to side” technique, with Vicryl 4-0.

**Figure 4:**
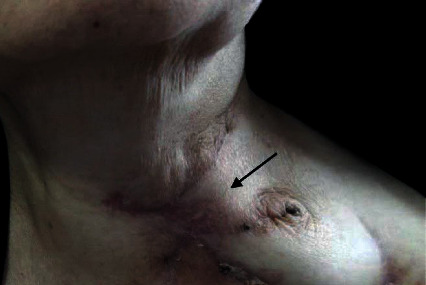
External view of the neck. Flash showing the supraclavicular flap pedicle, demonstrating a viable flap after 2 months of surgery.

**Figure 5:**
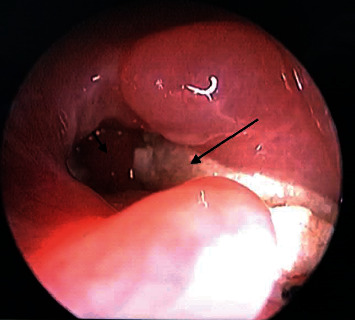
Examination of the pharyngeal entrance and supraclavicular flap using fiber-optic, after 2 months of surgery. The long arrow indicates supraclavicular flap and the short arrow indicates new pharyngeal lumen input.

During the abovementioned surgery, the larynx was preserved. Flap viability was evaluated using the flap pedicle which was visible and accessible from the anterior aspect of the neck (Figure 4). The postoperative feeding technique was via the nasogastric (NG) tube. The patient was discharged after decannulation on day 10 postsurgery. Three weeks after surgery, a barium swallow study was performed showing no evidence of anastomotic leakage. At the same time, the NG tube was removed and oral feeding was started. At week 5, complete movement of the true vocal cord (TVC) on the one side was observed. The patient was able to speak and consume nutrition orally without a tracheostomy. After successful initiation of oral feeding, the patient began to receive 30 sessions of adjuvant CRT. 1 year after surgery, no evidence of recurrence was observed (Figure 5).

Two key points should be considered when using this technique. First, deepithelialization was not performed on the base of the supraclavicular flap and it remained on the skin surface to monitor its postoperative viability. Second, the skin to skin suturing technique should be used for rolling various flaps, and deep sutures are to be avoided as they can lead to microcirculation dysfunction in distal parts.

## 3. Discussion

Although organ-preserving CRT is the treatment of choice for early stage HSCC (i.e., T1 and T2), primary surgery with reconstruction remains the favored therapeutic choice for patients with an advanced lesion due to CRT limitations, such as the long-term effects on laryngopharynx function and shorter survival in the event of salvage surgery [2, 10–12, 29, 30].

The PLE provides adequate oncologic control, with a considerable negative impact on patients' quality of life. Thus, conservation laryngeal procedures were developed to address the poor functional outcomes [31]. Partial laryngopharyngectomy and partial pharyngectomy are among these organ function-preserving procedures feasible in selected cases of HSCC [15]. Despite satisfactory oncological and functional outcomes, several disadvantages have been described for this procedure, including the surgical invasiveness, reconstruction procedure, postoperative management of tracheostomy, and relatively long rehabilitation periods [32].

In our previous study [28], we described a new “End to side” reconstruction technique in patients with SCC of pyriform sinus apex. 8 patients (stage III HSCC) underwent partial pharyngectomy, and the mass was resected with a convenient margin while the esophagus was preserved. The pharyngeal defect was reconstructed using the esophagus instead of a gastric pull-up and the mentioned innovative technique. The postoperative outcomes were superior compared to other techniques described in the literature.

In the present case report, we performed the “End to side” technique to reconstruct the hypopharyngeal defect with a rolled supraclavicular flap in a patient with an advanced SCC of the pyriform sinus (T3N1M0). In this particular patient, the larynx, medial wall of sinus pyriform, and postcricoid were not involved; thus, total laryngectomy could be avoided. This procedure enabled us to preserve the larynx and laryngeal orifice when either the lateral or medial wall of the pyriform sinus was involved. We rolled the easy harvested supraclavicular flap, placed it on the circumferential pharyngeal defect, and sutured it to the preserved larynx using the “End to side” technique to attach the rolled supraclavicular flap to proximal larynx similar to the technique applied in the previous study to attach the esophagus to the larynx. The main complications following conservative surgeries are aspiration and pharyngocutaneous fistula [33], which were not observed in our patient. With this technique, we achieved a clean margin, successful removal of the tracheostomy tube (at week 2), relatively short hospital stays (10 days), successful initiation of the oral diet without gastrostomy (at week 3), effective phonation and deglutition function, and even one normal TVC movement several weeks after surgery.

An important aspect of this method is that the laryngeal orifice should be reconstructed using oropharynx and postcricoid mucosa in such a way to create a defined lumen with the larynx and does not lead to laryngeal closure.

Few authors investigated the role of conservative surgeries in advanced HSCC. Jonnalagadda [22] used the tubed supraclavicular flap in 7 patients with advanced HSCC after total pharyngectomy. They concluded that the tubed supraclavicular flap could be a less morbid alternative for the reconstruction of circumferential hypopharyngeal defects.

Chung et al. [34] performed a laryngeal-preserving partial pharyngectomy technique in 58 patients with HSCC and reported a 5-year overall and disease-specific survival rates of 78% and 77.6%, respectively. In Holsinger et al.' study [15] of 30 patients with the advanced SCC of the pyriform sinus lateral wall undergoing partial lateral pharyngectomy, an immediate mortality rate, local recurrence rate, 1-year survival rate, and 5-year survival rate was 9%, 13%, 77.7%, and 40%, respectively. Joo et al. [17] performed partial hypopharyngectomy in 43 patients with HSCC with short-term morbidity and mortality of 0% and 7%, respectively. The recurrence, 5-year overall, and disease-specific survival rates were 35%, 63%, and 67%, respectively. Chung et al. [34] reported postoperative complications in 9 patients (6 cases of pharyngocutaneous fistula and 3 cases of laryngeal stenosis), and the oral diet was started with a mean duration of 26.1 and 31.4 days, while the percutaneous endoscopic gastrostomy tube (PEG) was required in 8 participants.

In Yoo et al.' study [33], 7 patients with SCC of the pyriform sinus (T1 or T2) without the involvement of apex underwent partial laryngopharyngectomy. The surgery resulted in good phonation and deglutition and mild aspiration as the most frequent postoperative complication. The long-term evaluation revealed 1 case of regional recurrence and one distant metastasis with no evidence of recurrence at the primary site.

Lim et al. [14] evaluated the role of the larynx-preserving partial pharyngectomy via the lateral pharyngotomy in 23 patients with small HSCC (T1 or T2). The procedure was associated with a 2-year and 5-year survival rate of 77% and 61% and a recurrence rate of 39%. The ultimate cure rate of the primary tumor was 87%. Successful decannulation, proper oral diet, and acceptable postoperative speaking function were observed in 95% of cases. The authors suggested this surgical technique as a feasible procedure in patients with small HSCC. Conversely, Czaja and Gluckman [35] suggested a wider resection when facing early HSCC as partial pharyngectomy was associated with a higher locoregional recurrence and thus decreased survival rate compared to more extensive procedures. The high frequency of submucosal spread combined with multiple skip lesions could be regarded as the reason beyond the high rate of local recurrence [14]. This implies the importance of the CRT after conservative surgeries as both Lim et al. [14] and Frank et al. [36] reported more cases of recurrent disease with surgery alone compared to surgery followed by postoperative CRT.

Another larynx-preserving technique in patients with advanced pyriform sinus is the supracricoid hemilaryngopharyngectomy [37]. According to Papacharalampous et al. [38], this technique was associated with 5-year survival and recurrence rate of 55.6% and 16.7%, respectively, with neither participant requiring total laryngectomy.

Compared to similar articles, our suggested technique not only could preserve the esophagus and larynx with good postoperative speech and swallowing function but also was associated with acceptable oncologic results and no recurrence after 1 year.

Reconstruction of the hypopharyngeal defect is a challenging issue for the surgeons [18]. The ideal technique for hypopharyngeal reconstruction is a safe, 1-stage procedure with a high success rate of tissue transfer, low donor-site morbidity, and low fistula and stenosis rates leading to early restoration of function (speech and swallowing) and a high tolerance for postoperative irradiation [7, 18, 39].

Recently, the supraclavicular fasciocutaneous flap has been popularized as the reconstructive method for a variety of head and neck defects [40–42]. The supraclavicular flap is a thin and pliable, hairless, versatile, vascularized, and regional flap, with acceptable cosmetic (color and texture match) and functional outcomes for head and neck oncologic defects [43–45]. This flap is well tolerated by the patient, with minimal donor-site morbidity and good viability of the recipient site [46].

This technique has several advantages over free tissue transfer. As it is adjacent to the donor site, it matches the recipient site, properly [43]. A supraclavicular flap can be harvested quickly as the mean time spent to harvest the flap is less than 1 hour, reducing both donor and recipient site morbidities [7, 45, 47]. Furthermore, it does not require microvascular surgery and thus, microvascular experience [47]. Previous CRT and radical neck dissection, poor nutrition, significant smoking history, and previous tracheotomies increase flap failure therefore should be investigated before surgery.

## 4. Conclusions

Laryngeal-preserving partial pharyngectomy and hypopharyngeal reconstruction using the rolled supraclavicular flap via the “End to side” technique could lead to good oncological and functional outcomes in selected cases of pyriform sinus SCC.
